# Llama-Derived Single Domain Antibodies to Build Multivalent, Superpotent and Broadened Neutralizing Anti-Viral Molecules

**DOI:** 10.1371/journal.pone.0017665

**Published:** 2011-04-01

**Authors:** Anna Hultberg, Nigel J. Temperton, Valérie Rosseels, Mireille Koenders, Maria Gonzalez-Pajuelo, Bert Schepens, Lorena Itatí Ibañez, Peter Vanlandschoot, Joris Schillemans, Michael Saunders, Robin A. Weiss, Xavier Saelens, José A. Melero, C. Theo Verrips, Steven Van Gucht, Hans J. de Haard

**Affiliations:** 1 Department of Biology, Cellular Architecture and Dynamics, University of Utrecht, Utrecht, The Netherlands; 2 Division of Infection and Immunity, Medical Research Council/University College London Centre for Medical Molecular Virology, University College London, London, United Kingdom; 3 Medway School of Pharmacy, University of Kent, Chatham Maritime, Kent, United Kingdom; 4 Communicable and Infectious Diseases, Rabies Laboratory - Scientific Institute of Public Health, Brussels, Belgium; 5 Ablynx NV, Gent, Belgium; 6 Department for Molecular Biomedical Research, VIB, Gent, Belgium; 7 Department for Biomedical Molecular Biology, Gent University, Gent, Belgium; 8 Department of Pharmaceutics, Utrecht Institute for Pharmaceutical Sciences (UIPS), Utrecht University, Utrecht, The Netherlands; 9 Centro Nacional de Microbiología and CIBER de Enfermedades Respiratorias, Instituto de Salud Carlos III, Madrid, Spain; French National Centre for Scientific Research, France

## Abstract

For efficient prevention of viral infections and cross protection, simultaneous targeting of multiple viral epitopes is a powerful strategy. Llama heavy chain antibody fragments (VHH) against the trimeric envelope proteins of Respiratory Syncytial Virus (Fusion protein), Rabies virus (Glycoprotein) and H5N1 Influenza (Hemagglutinin 5) were selected from llama derived immune libraries by phage display. Neutralizing VHH recognizing different epitopes in the receptor binding sites on the spikes with affinities in the low nanomolar range were identified for all the three viruses by viral neutralization assays. By fusion of VHH with variable linker lengths, multimeric constructs were made that improved neutralization potencies up to 4,000-fold for RSV, 1,500-fold for Rabies virus and 75-fold for Influenza H5N1. The potencies of the VHH constructs were similar or better than best performing monoclonal antibodies. The cross protection capacity against different viral strains was also improved for all three viruses, both by multivalent (two or three identical VHH) and biparatopic (two different VHH) constructs. By combining a VHH neutralizing RSV subtype A, but not subtype B with a poorly neutralizing VHH with high affinity for subtype B, a biparatopic construct was made with low nanomolar neutralizing potency against both subtypes. Trivalent anti-H5N1 VHH neutralized both Influenza H5N1 clade1 and 2 in a pseudotype assay and was very potent in neutralizing the NIBRG-14 Influenza H5N1 strain with IC_50_ of 9 picomolar. Bivalent and biparatopic constructs against Rabies virus cross neutralized both 10 different Genotype 1 strains and Genotype 5.

The results show that multimerization of VHH fragments targeting multiple epitopes on a viral trimeric spike protein is a powerful tool for anti-viral therapy to achieve “best-in-class” and broader neutralization capacity.

## Introduction

Viruses are a continuous threat to humans, exemplified by the recent appearance of the 2009 pandemic H1N1 influenza virus. Because of the genetic variability of RNA viruses, they are difficult to control by prophylactic or anti-viral therapy. Vaccines need to induce a neutralizing immune response against highly conserved epitopes to be effective, but very limited success has been obtained so far. Several very potent anti-viral compounds have been developed for treatment of for instance HIV, Hepatitis B and influenza infections, but their use have rapidly been followed by the appearance of drug-induced escape mutants [1], [2].

For many enveloped viruses, entry into target cell depends on fusion of the viral and cell membranes, driven by the interaction of viral glycoproteins with the target cell membrane. In this study, we evaluated three different negative strand RNA viruses with trimeric envelope proteins, Fusion protein (F protein) of Respiratory Syncytial Virus (RSV), H5 hemagglutinin of H5N1 avian Influenza and Rabies glycoprotein (G protein). RSV is the major cause of lower respiratory infection and hospitalization of infants and young children and the current prophylactic treatment with the monoclonal antibody Synagis is restricted to infants that are premature or have heart or lung disease. Influenza H5N1 (avian flu) is highly pathogenic and virulent and is spread from poultry to humans, causing viral pneumonia that may be fatal. There is no current cure, but vaccines and neutralizing antibodies are being developed. Rabies is also a virus spread from animals to humans and causes acute encephalitis which is fatal if post-exposure prophylaxis is not administered before the virus has infected the brain. All three viruses cause severe infections in humans and even though neutralizing antibodies are available for Rabies and RSV, there is a need for alternative and improved antiviral therapy.

Llama-derived single domain VHHs have proven to be powerful viral neutralizers [3], [4], [5], [6]. The single chain nature of the VHHs allows construction and production of multimeric molecules using the same or different VHH building blocks [7], [8]. Single domain molecules (VHH) are the antigen binding, variable part of heavy chain only antibodies. These heavy-chain antibodies are devoid of the light chain and found in members of the *Camelidae* family, such as the llama [9]. VHHs are small (12–15 kDa), stable molecules with improved solubility and similar affinities as conventional antibodies [10]. These properties make them promising molecules for prophylactic and therapeutic purposes.

In this study, we demonstrate that the formatting flexibility of the VHH allows the generation of anti-viral molecules with low picomolar neutralizing potencies, up to 4,000-fold better than the monovalent VHH, and broadened neutralizing activities, likely overcoming the chance of virus escaping neutralization. The latter improvement was obtained by either fusing VHH recognizing different epitopes, but also by fusing multiple copies of the same VHH. Similar results were obtained with VHHs against the trimeric spike proteins of all three viruses.

These data demonstrate the general applicability of VHHs for construction of highly potent anti-viral molecules for treatment of viral infections.

## Results

### Isolation of viral spike protein specific VHH

Two llamas per viral target were successfully immunized with the following antigens; RSV F_TM-_ protein, which is a recombinant trimeric membrane anchor less form of the fusion protein of human Respiratory Syncytial Virus (Long strain, subgroup A), recombinant trimeric H5N1 Hemagglutinin (H5, A/Vietnam/1203/2004) and Inactivated Rabies Vaccine Mérieux HDCV (genotype 1, Wistar strain of the Pitman Moore virus).

Selections were performed using recombinant RSV F_TM-_ protein, recombinant H5 Hemagglutinin and Rabies genotype 1, PV glycoprotein glycoprotein. Binding phage were eluted from the antigen by unspecific or competitive elution using an excess of viral neutralizing antibodies for RSV (Synagis) and Rabies (Mab 8-2). After two rounds of selections, individual clones were isolated, VHH were produced, purified and screened for binding to the viral antigens in ELISA and competition with monoclonal antibodies or in the case of Influenza H5N1, the sialylglycoprotein fetuin. For RSV, 188 clones were investigated for binding to the F_TM-_ protein revealing that 79% were interacting. Only 30% binders were identified for H5 hemagglutinin, while 50% binders were identified for Rabies G protein. The binding clones were tested for competition with the antibodies (Synagis or mAb 8-2) or fetuin and for all the viral targets competitors were identified that were sequenced and further analyzed for neutralization properties.

By fusing RSV specific VHH, a total of 3 bivalent and 4 biparatopic (i.e bispecific) constructs were made. Two bivalent and two trivalent Influenza H5N1 constructs and four bivalent and four biparatopic anti-rabies VHH constructs were made. The linker lengths (Gly_4_/Ser) were between 9 and 35. The production yields for the multimeric constructs were between 0.5 mg/L to 1 mg/L after IMAC purification as compared to monovalent VHH, with 3–10 fold higher yields.

### Neutralization and cross-protection of RSV by VHH

**Figure 1:**
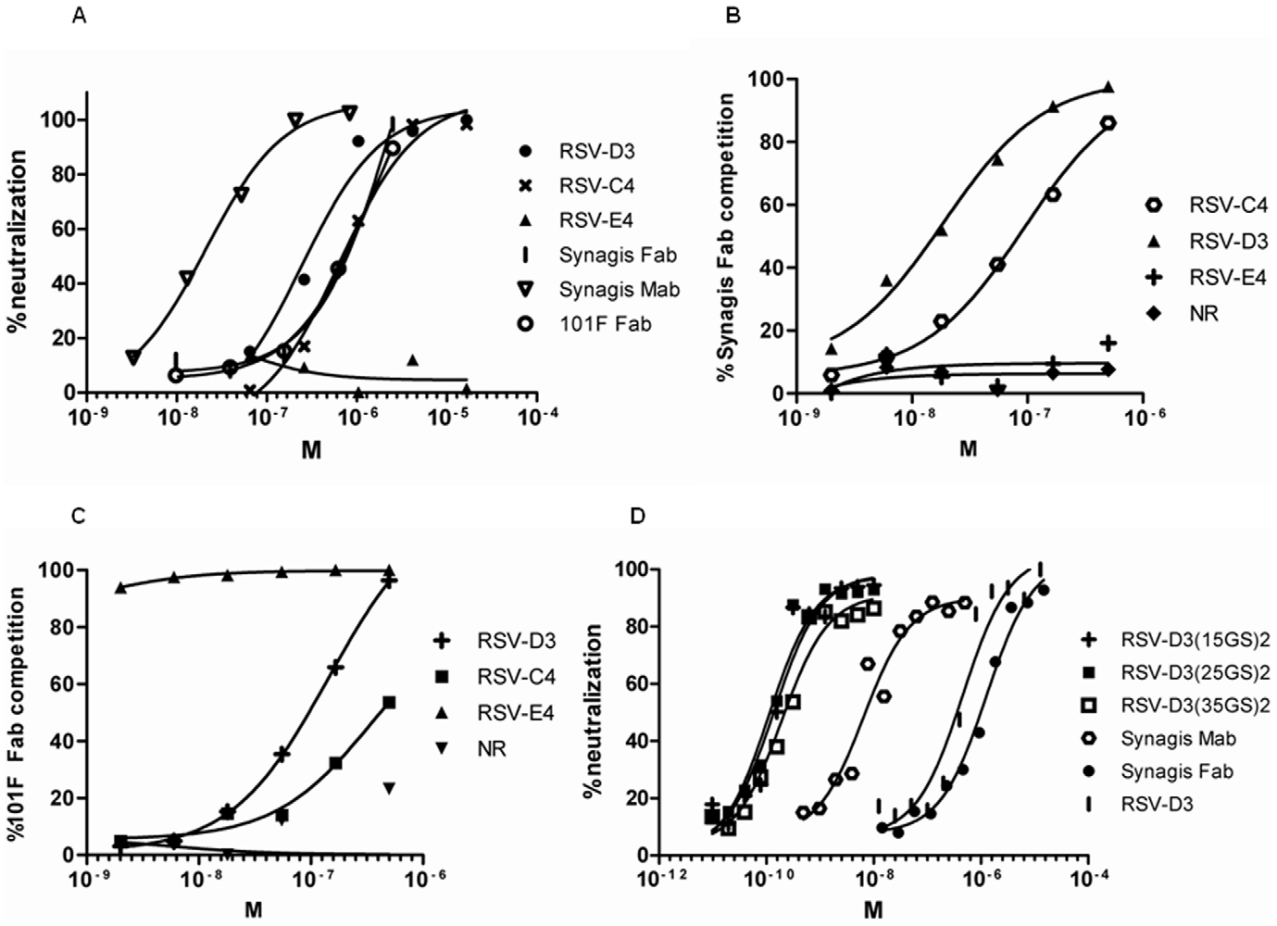
Microneutralization and antibody competition of RSV-specific VHH. Monovalent VHH neutralizing RSV Long strain, subtype A. Neutralization is expressed in percentage as compared to controls with irrelevant VHH, plotted against the concentrations of VHH in molar (M) (A). Anti-RSV VHH competing with 3 nM Synagis Fab for binding to immobilized RSV F protein, presented in percentage of competition as compared to controls with no VHH or irrelevant VHH. NR is an irrelevant control VHH against H5N1. Binding of Synagis Fab was detected as described in the text and absorbance was read at 450 nm (B). Anti-RSV VHH competing with 3 nM 101F Fab for binding to immobilized RSV F protein, presented in percentage of competition as compared to controls with no VHH or irrelevant VHH. NR is an irrelevant control VHH against H5N1. Binding of 101F Fab was detected as described in the text and absorbance was read at 450 nm. (C). Bivalent RSV-D3 constructs with linker lengths from 15-35GS neutralizing RSV Long strain, subtype A. Neutralization is expressed in percentage, as compared to controls with irrelevant VHH and plotted against the concentrations of VHH in molar (M) (D). All experiments were repeated two times and the figure represents one experiment.

Based on the screenings of the selected clones in binding ELISA and competition assays, twelve purified VHHs were tested in an *in vitro* micro-neutralization assay for neutralization of the RSV Long strain (subgroup A). Two of the VHH (RSV-D3 and RSV-C4) neutralized RSV Long strain. RSV-D3 was the most potent with an IC_50_ of 250 nM (Fig. 1A). IC_50_ for both Synagis Mab and Fab were in the range of what has previously been reported [11]. RSV-D3 and RSV-C4 VHH did not have any neutralization effect on RSV B1 strain (subgroup B). However, there was a minor effect by monovalent RSV-E4, which did not neutralize the RSV Long strain (data not shown).

For further characterization of the neutralizing VHHs, ELISA based competition assays were performed on immobilized RSV F_TM-_ protein, using Fab fragments derived from two different neutralizing monoclonal antibodies, Synagis (humanized version of mouse Mab 1129, recognizing antigenic site II) and 101F (recognizing antigenic site IV–VI), binding different epitopes on the F protein [12], [13], [14]. VHHs RSV-D3 and RSV-C4 competed with Synagis Fab and RSV-E4 competed with 101F Fab for binding to RSV F_TM-_ protein (Fig. 1B and C). RSV-D3 and RSV-C4 also showed some competition at high concentrations (µM) with 101F Fab, indicating the epitope recognized by these VHH is overlapping to some degree, but most binding parts are in the antigenic site II (Fig. 1C). These results were confirmed in independent experiments using both the Fabs and the monoclonal antibodies of Synagis and 101F (data not shown). The Fab competitions were also confirmed on immobilized inactivated RSV (data not shown).

**Figure 2:**
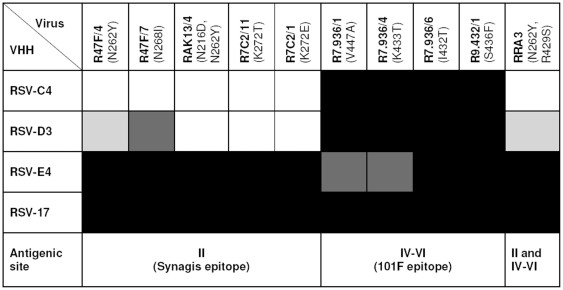
Binding of VHH in ELISA to RSV escape mutants using HEp-2 cells infected with the indicated viral strains as substrate. Absorbance results were normalized for reactivity on the reference virus strain (Long wild type) strain as well as on the control VHH RSV-C7 (RSV binder, but non-competitor to Synagis and 101F). (▪)>75% reactivity compared to RSV-C7, (▪) 50–75% reactivity compared to RSV-C7, (▪) 50–25% reactivity compared to RSV-C7, (□) <25% reactivity compared to RSV-C7. Binding was confirmed in two independent experiments.

The epitopes recognized by VHHs directed against RSV F protein were further investigated by testing their reactivity in ELISA with previously described RSV escape mutants [15]. Absorbance results were normalized for reactivity on the reference virus strain (Long wild type) as well as on the control RSV-C7 (RSV F_TM-_ protein binder, but non-competitor). VHH RSV-D3 and RSV-C4 were found to be sensitive to typical mutations in antigenic site II, confirming the competition pattern with Synagis, while VHH RSV-E4 was sensitive to mutations in the antigenic site IV–VI, confirming the competition results with 101F (Fig. 2). RSV-C4 and RSV-D3 bound to the same mutants with some small differences, possibly due to differences in affinity or recognition of overlapping epitopes. RSV-D3 showed a weak binding to RRA3, a double mutant with changes in both site II and IV–VI.

Affinities of the monovalent VHHs selected against RSV were measured by Surface Plasmon Resonance and were in the low nanomolar range (Table 1). VHH RSV-E4, bound to RSV F protein with an affinity (*K_D_*) of 0.45 nM, which was slightly better than the Mab Synagis with a *K_D_* of 0.64 nM and about 15-fold better than the Fab fragment of Synagis.

**Table 1:** Affinities of monovalent VHH against recombinant Influenza H5 and RSV F protein measured by Surface Plasmon Resonance.

sample	antigen	*k_on_* (M^−1^ s^−1^)	*k_off_* (s^−1^)	*K_D_* (nM)^a^
Infl-C8	Influenza H5	4.99×10^5^	4.95×10^−3^	9.91
Infl-B12	Influenza H5	2.14×10^5^	6.45×10^−3^	30.1
RSV-D3	RSV F_TM-_	9.89×10^5^	9.14×10^−3^	1.78
RSV-C4	RSV F_TM-_	1.48×10^6^	2.64×10^−3^	9.24
RSV-E4	RSV F_TM-_	4.64×10^5^	2.09×10^−4^	0.45
Synagis Mab	RSV F_TM-_	2.77×10^5^	1.78×10^−4^	0.64
Synagis Fab	RSV F_TM-_	1.69×10^5^	5.05×10^−4^	2.99

a Equlibrium dissociation constant *K_D_* (*k_off_*/*k_on_*), association rate constant *k_on_* and dissociation constant *k_off_* determined by Surface Plasmon Resonance.

Next, we investigated if linking two identical RSV-D3 VHHs (bivalent RSV-D3) with flexible Gly_4_/Ser linkers of different lengths could enhance the neutralization potency of VHHs. Remarkably, bivalent RSV-D3 VHHs could neutralize RSV Long strain about 4000-fold better than the monovalent RSV-D3 VHH. All three bivalent VHH neutralized the RSV Long strain better then Synagis Mab and Synagis Fab with IC_50_'s of approximately 0.1 nM compared to 6.5 nM for Synagis Mab and 1.3 µM for Synagis Fab (Fig. 1D and Table 2). There was no significant difference in neutralization capacity of RSV Long strain for the bivalent RSV-D3 constructs with different linker lengths (15, 25 or 35 GS linkers). All experiments were run two independent times comparing with the controls Synagis Fab or mAb and the monovalent RSV-D3 showing the same fold difference in neutralization as compared to the monovalent RSV-D3.

**Table 2:** Microneutralization of RSV subtype A and B.

Microneutralization, IC_50_ (nM)			
	RSV Long (A)		RSV B1 (B)
**monovalent VHH**			
RSV-C4	640		n.d.^b^
RSV-D3	460±149^d^		>1000
RSV-E4	No effect		>1000
RSV-D3+RSV-E4	n.d.		>1000
**Bivalent VHH**		**Potency increase** a	
RSV-D3(15GS)_2_	0.14	3285	103
RSV-D3(25GS)_2_	0.11	4181	n.d
RSV-D3(35GS)_2_	0.19	2421	n.d
**Biparatopic VHH**			
RSV-D3/E4(9GS)	6	37	1.8
RSV-D3/E4(15GS)	5.2	43	n.d.
RSV-D3/E4(25GS)	18	12	n.d
RSV-E4/D3(9GS)	100	2	29
**Mab/Fab**			
Synagis Mab	6.5±10	200^c^	2.1
Synagis Fab	1300±990	-	n.d.
101F Fab	1500	-	101

a increased potency against RSV Long (A) compared to the monovalent RSV-D3 VHH.

b not determined.

c increased potency compared to Fab.

d SD of two independent experiments with the controls.

To target two different epitopes on the RSV F protein, biparatopic VHH construct containing two different VHHs were generated with linker lengths of 9, 15 or 25 GS residues. The biparatopic VHH RSV-D3/E4 contained RSV-D3 and RSV-E4 VHH, which respectively recognized the Synagis-like epitope and the 101F-like RSV epitope. The biparatopic constructs were investigated for cross neutralization of both RSV Long strain and RSV B1 strain.

The two biparatopic VHHs, RSV-D3/E4 VHH having linkers of either 9 or 15 residues, had comparable neutralization potency as the Synagis Mab (IC_50_ of 6.5 nM) and were about 40-fold better in neutralizing the Long strain (subtype A) and RSV-D3/E4(9GS) was more than 500-fold better for the B1 strain as compared to the monovalent VHHs. There was no significant difference in neutralization potency of the RSV Long strain between the two biparatopic VHHs RSV-D3/E4(9GS) and RSV-D3/E4(15GS) (IC_50_ of 6 nM and 5.2 nM, respectively) (Table 2). The biparatopic construct with a 25 GS linker had an IC_50_ of 18 nM for RSV Long strain, about 3-fold lower than the constructs with the shorter linkers, 9 or 15 GS. The biparatopic constructs were not as potent in neutralization of RSV Long strain as the bivalent RSV-D3.

We further investigated if neutralization of the RSV B1 strain was achieved with the biparatopic constructs and if the order of the VHH made a difference for the potency. The biparatopic constructs with RSV-D3 in the N-terminal and RSV-E4 in the C-terminal position and a linker length of 9 GS (RSV-D3/E4) was about 15-fold more potent in neutralizing the RSV B1 strain as compared to the fusion protein with the VHH in the opposite direction (RSVE4/D3), IC_50_ of 1.8 nM compared to 29 nM (Table 2). There was a slightly better neutralizing effect when combining RSV-E4 (5 nM) and RSV-D3 (5 nM) in an equimolar ratio compared to monovalent RSV-E4 (10 nM), but not comparable to the potency of the biparatopic constructs.

### Neutralization and cross clade protection of VHH against Influenza H5N1

**Figure 3:**
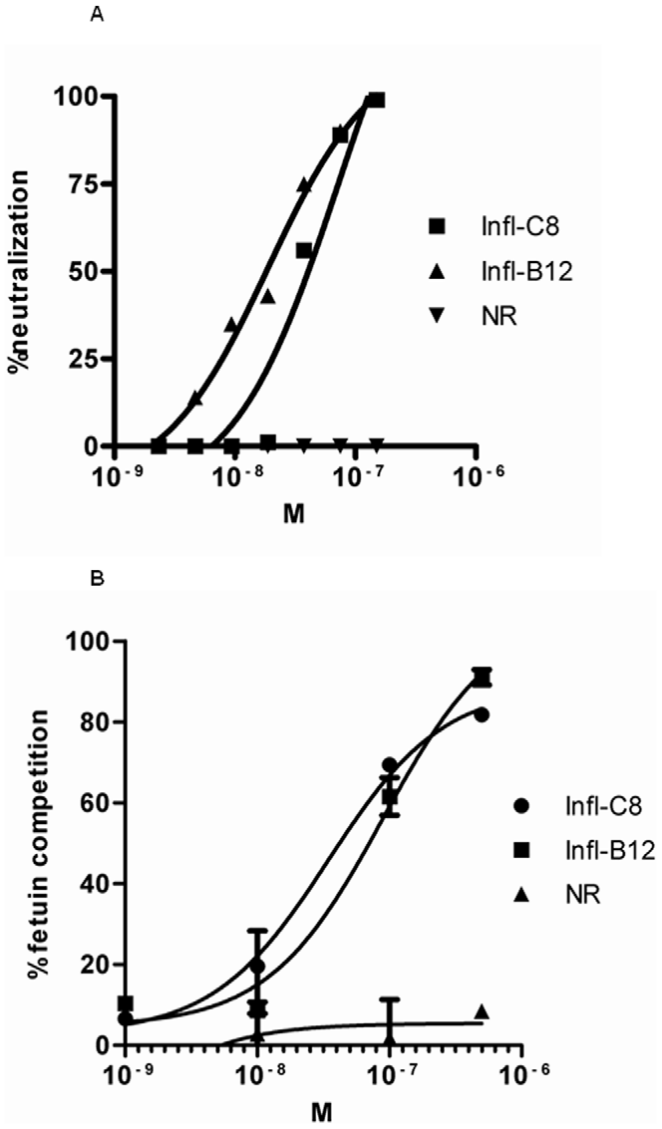
Neutralization and fetuin competition of H5N1-specific VHH. Monovalent VHH neutralizing Influenza H5N1, A/Vietnam/1203/04 in a pseudotyped neutralization assay. Neutralization (geometric mean of duplicates) is expressed in percentage compared to the virus only and plotted against the concentrations of VHH in molarity (M) (A). Dilutions of monovalent VHH against Influenza H5 in duplicates, competing with 10 µg/ml immobilized fetuin for binding to biotinylated H5, expressed as percentage competition. Plotted against concentrations of VHH in molarity (M). NR is an irrelevant VHH binding to RSV. Detection of biotinylated H5 as indicated in text. Standard deviation (SD) indicated by bars (B).

The neutralizing capacity of VHH against Influenza H5 A/Vietnam/1203/04 (clade 1) viruses, were evaluated in the MLV(H5) pseudotyped neutralization assay [16]. Two of 28 tested VHHs (Infl-C8 and Infl-B12), selected on A/Vietnam/1194/04, neutralized the pseudotyped A/Vietnam/1203/04 virus (Fig. 3A). The neutralization was run in duplicates and the graph represents the geometric means. Both VHH competed with sialylglycoprotein fetuin, which binds the receptor binding site of hemagglutinin 5 (Fig. 3B). The VHHs had affinities to hemagglutinin 5 of clade 1, A/Vietnam/1194/04 in the nanomolar range (9.9 nM and 30 nM for Infl-C8 and Infl-B12, respectively) (Table 1). VHHs Infl-C8 and Infl-B12 neutralized A/Vietnam/1194/04 as effectively as A/Vietnam/1203/04. No synergistic effect was observed when mixing the neutralizing VHH in an equimolar ratio, only an additive effect (data not shown).

Based on the neutralization data from the VHH against Influenza H5 clade 1 pseudotype strains (A/Vietnam/1203/04 and A/Vietnam/1194/04) and epitope recognition, bivalent and trivalent constructs were generated, by fusing individual VHH domains with Gly_4_/Ser linkers. The majority of the tested bivalent and trivalent VHH constructs had a significantly higher neutralizing potency than the corresponding monovalent VHH against all four clades of H5N1 tested, with the bivalent and trivalent constructs based on Infl-C8 being the best neutralizers (Table 3). Bivalent Infl-C8 constructs against H5N1 showed no difference in neutralization potency for the two clade 1 hemagglutinins A/Vietnam/1194/04 and A/Vietnam/1203/04. The neutralizing potency of the trivalent Infl-C8 against various clades of Influenza H5N1 was significantly increased compared to monovalent VHH against all the H5N1 clades tested. The trivalent VHH, Infl-C8 with a 10 GS linker had a 75-fold increased potency compared to the corresponding monovalent VHH in neutralizing the A/Vietnam clades. Trivalent Infl-C8 with a 20 GS linker had a slightly lower potency than the 10 GS linker construct (Table 3). All experiments were run in duplicates and presented as IC_50_ of the geomeotric mean of the monovalent VHH. Remarkable is the jump in potency for the bivalent Infl-C8 construct with a 10 GS linker for the clade 2.2 strains, whereas the same construct with 20GS linker has a somewhat lower neutralization capacity.

**Table 3:** Neutralization of Influenza H5N1 virus.

Neutralization of Influenza H5N1 virus IC_50_ (nM)^a^					
H5N1 subtype	Infl-C8(monovalent)	Infl-C8(9GS)_2_(bivalent)	Infl-C8(15GS)_2_(bivalent)	Infl-C8(10GS)_3_(trivalent)	Infl-C8(20GS)_3_(trivalent)
A/Vietnam/1194/04 (Clade 1)	75	<1	<1	<1	10
A/Vietnam/1203/04 (Clade 1)	75	<1	<1	<1	10
A/turkey/Turkey/1/05 (Clade 2.2)	120	120	120	3	75
A/Bar-headed goose/Qinghai/1A/05 (Clade 2.2)	50	50	50	7	40
A/Whooping swan/Mongolia/244/05 (Clade 2.2)	>150	150	150	18	75
A/Anhui/1/05 (Clade 2.3.4)	>150	9	5	5	75
A/chicken/Korea/ES/03 (Clade 2.5)	No effect	<1	<1	<1	15
A/NIBRG-14^b^	7	n.d.^c^	0.009	0.003	n.d.

a IC_50_ of VHH neutralizing pseudotyped MLV(H5) virus infection as compared to pseudotyped virus-only control and cells only. IC_50_ calculated from duplicates as geometric mean.

b IC_50_ of VHH in a microneutralization assay on MDCK cells.

c not determined.

The neutralization potency and cross protection by the bivalent and trivalent VHH was further confirmed in a microneutralization assay using the NIBRG-14 Influenza H5N1 strain on MDCK cells. The potencies for the neutralization was in the picomolar range (IC_50_ 9 and 3 pM for the bi- and trivalent VHH, respectively), which is about 1,000-fold more potent than the previously published monoclonal antibody CR6261 (IC_50_ of around 3–4 nM) [17]. No cross-neutralization of PR8 (H1N1) or X47 (H3N2) virus was observed.

Finally, a hemagglutination inhibition assay (HAI) was performed to investigate if the Infl-C8 constructs sterically blocked the attachment of Influenza H5N1 virus, NIBRG-14, to chicken erythrocytes (RBC). The minimal concentration of bivalent and trivalent Infl-C8 inhibiting hemagglutination of RBC by NIBRG-14 virus was 2 nM, as compared to the monovalent Infl-C8, which inhibited hemagglutination at 156 nM.

### Neutralization of divergent Lyssavirus genotypes

VHH selected against the Rabies genotype 1, PV glycoprotein, were examined for cross-neutralization of different genotype 1 lyssaviruses (three laboratory strains and seven street isolates) and one genotype 5 lyssavirus (European bat lyssavirus-1, EBLV-1). Human cases of Rabies (>99%) are caused by genotype 1 lyssaviruses [18]. EBLV-1 circulates in certain species of bats (mainly *Eptesicus serotinus*) in Europe [19].

**Figure 4:**
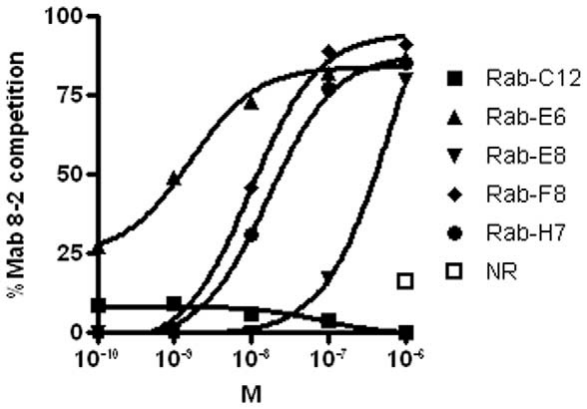
Antibody 8-2 competition of anti-Rabies VHH. Serial dilutions of VHH against Rabies G protein competing with 4 nM Mab 8-2 for binding to immobilized G protein. Detection of Mab 8-2 as indicated in text. Competition is expressed in percentage compared to the Mab 8-2 bound by immobilized G protein and plotted against the concentrations of VHH in molarity (M). NR was included at 1 µM only. The assay was repeated two times and this figure represents one run.

In general, VHH which neutralized the prototype CVS-11 strain (genotype 1), also neutralized most other genotype 1 viruses, including street isolates (Table 4). Rab-E8 was not neutralizing the ERA strain, but was also able to neutralize the divergent genotype 5 EBLV-1 strain, which also Rab-H7 neutralized (Table 5). The neutralization pattern of the different VHH together with the results from the competition assays with Mab 8-2 indicated recognition of antigenic site IIa [20] for all but Rab-C12, which seemed to recognize a different epitope (Figure 4). Although Rab-E6, Rab-H7, Rab-E8 and Rab-F8 recognized a partially overlapping epitope, their fine specificity differed, because only Rab-E8 and Rab-H7 cross-reacted with EBLV-1 (genotype 5) (Table 5).

**Table 4:** Neutralization of Genotype 1 Rabies strains determined by Rapid Fluorescent Focus Inhibition Test (RFFIT) with cell grown-virus.

Neutralization of Rabies genotype 1 strains IC_50_ (nM)			
	ERA (Evelyn-Rotnycki-Abelseth)	CB-1 (Chien Beersel)	7 street strains^b^
Mab 8-2	22.6^a^	13.0	6/7
Rab-F8	94	18.1	7/7
Rab-E8	>3871	18.3	nd
Rab-E6	14.4	4.17	6/7
Rab-H7	25.6	1.05	5/7
Rab-C12	6.63	4.86	4/7
NR1 (anti-RSV)	>5956	>5956	0/7
NR4 (anti-RSV)	>4839	>4839	0/7

a Mean IC_50_ (nM) of triplicates.

b Tissue Culture Infectious Dose 50%, which corresponds with the dilution of the infected brain suspension – VHH mixture which yields 50% infection of neuroblastoma cells of isolates: 9912CBG (dog, Cambodia), 9147 FRA (fox France), CVS (strain IP13), 9722 POL (raccoon dog, Poland), 8740 THA (Human, Thailand), 070591C (dog, Ivory coast), 9009 NIG (dog, Niger). Neutralization considered if a minimum of 100-fold reduction of virus infectivity in the brain was observed after preincubation with antibody (Mab 8-2) or VHH compared to a control VHH (NR4 or NR1 anti-RSV) (#neutralization/#total).

**Table 5:** Neutralization of Lyssavirus determined by Rapid Fluorescent Focus Inhibition Test (RFFIT).

Neutralization of rabies virus IC_50_ (nM)^a^				
	CVS-11 (genotype 1)		EBLV-1 (genotype 5)	
sample	nM IC_50_		nM IC_50_	
Mab 8-2	0.25		0.12	
Rab-C12^b^	7.55		>9529	
Rab-E6	13.66		>4913	
Rab-H7	191.4		586	
Rab-E8	248.9		520	
Rab-F8	324.9		>1191	
NR (irrelevant anti-RSV)	>5956		>5956	

		Increased potency^c^		Increased potency
Rab-C12_2_	3.74	2	>4167	>2
Rab-H7_2_	2.09	92		
Rab-E8_2_	3.28	76		
Rab-F8_2_	1.79	181		
Rab-E6/H7	0.26	394	237	>12
Rab-E8/H7	0.14	1572	3.76	147
Rab-E8/C12	3.69	35		
Rab-H7/F8	0.33	782	108	>8
NR_2_ (irrelevant anti-RSV)	>725	0		

a Mean IC_50_ of triplicates.

b Monovalent and bivalent/biparatopic with 15GS linkers run at two different time points, but standards OIE (canine reference serum) 0.5 IU/ml, WHO human reference serum 0.5 IU/ml and WHO human reference serum 6.0 IU/ml were included in all experiments as controls.

c Potency compared to monovalent VHH (nM). For the biparatopic constructs, potency increase is based on the mean (nM) of the two monovalent VHH included in the constructs.

Based on the data from the neutralization and the competition assays, biparatopic and bivalent constructs were generated with a Gly_4_/Ser linker of 15 residues. The majority of the tested bivalent and biparatopic VHH constructs had a significantly higher neutralizing potency than the corresponding monovalent VHH, both for CVS-11 and EBLV-1 (Table 5). Exceptions were constructs containing Rab-C12, bivalent or biparatopic, which did not, or in some cases, only moderately improve neutralization. As a bivalent Rab-C12, it neutralized 2 street isolates that were not neutralized by the monovalent Rab-C12. Compared to the monovalent VHH, the biparatopic combination Rab-E8/H7 was more than 1,500-fold more potent that the corresponding VHHs (Rab-E8 and Rab-H7) in neutralizing CVS-11 with an IC_50_ of 0.14 nM and about 150-fold better for EBLV-1 (Table 5). Fusion of Rab-E6 with Rab-H7 (Rab-E6/H7) improved neutralization of CVS-11 with almost 400-fold and EBLV-1 with more than 10-fold compared to the monovalent VHH (Table 5). No synergy was observed when mixing the neutralizing VHH in equimolar ratio together, only an additive effect (data not shown).

## Discussion

In this study, neutralizing VHHs were generated against trimeric glycoproteins of three different viruses and the potency and broadness was greatly improved by multimerization of VHH. The trimeric spike proteins of the three targeted viruses are functionally different, implying a different mechanism of action for the multivalent VHH constructs for neutralization of the viruses. Rabies virus and Influenza virus both use their trimeric spike proteins, G protein and hemagglutinin, for attachment to the host cell to allow cell invasion. RSV on the other hand uses its glycoprotein for attachment to the host cell and then the F protein for fusion to the host cell membrane after a conformational change [12], [13], [14]. Synagis (Palivizumab) blocks the fusion of RSV to its host cell membrane and is the only clinically used monoclonal antibody against an infectious disease [21].

Viral neutralizing monovalent VHH, with potencies in the micromolar range, were generated by immunization of llamas with recombinant trimeric envelope spike proteins or with (inactivated) virus from three different viruses, RSV, Rabies virus and highly pathogenic Influenza H5N1 virus. The single domain nature of the VHHs allows building of multimeric constructs by fusion of individual VHHs, either recognizing the same (bi/trivalent) or different epitopes (biparatopic).

The best improvements in neutralizing potency were observed for the RSV neutralizing VHH, RSV-D3. When engineered into a bivalent construct, it was approximately 4,000-fold more potent than the monovalent fragment, which already had a 2.6-fold better IC_50_ than the Fab fragment from Synagis. The bivalent RSV-D3 VHH outperformed the Synagis antibody in the microneutralization assay (44-fold better) with IC_50_ in the picomolar range. Synagis had a 180-fold better potency than the derived Fab due to bivalent binding, indicating that the formatted VHH RSV-D3, which competes with and therefore recognizes the same region as Synagis, must achieve its greatly increased potency by a different avid interaction. We propose that the bivalent VHH RSV-D3 can bind both intramolecular to two of the three F-proteins in a spike. Intramolecular binding has been described for TRAP molecules, like VEGF TRAP, in which the extracellular domains (ECD) of VEGF receptors were fused to the Fc of an IgG [22]. VEGF TRAP forms soluble 1 to 1 complexes by intramolecular binding of the two ECDs with the dimeric VEGF molecule, giving picomolar affinities and potencies in bioassays.

Although resistance to Synagis does not yet appear to be a clinical issue, wider use may increase this potential [23]. Targeting only one viral epitope with an antibody is a risk, because the virus can escape relatively easy neutralization by adopting mutations within the epitope recognized by the mono-specific antibody, whereas this is more difficult for two or more targeted epitopes. One way to overcome this is to give cocktails of antibodies. This has been done for HIV-1 and Rabies and increased neutralization potency as well as increased cross protection was observed, but only with an additive effect [24], [25]. In this study we demonstrated for both RSV and Rabies virus that biparatopic VHHs not only have increased neutralization activity as compared to their monovalent counterparts, but also display a broader cross subtype neutralization activity. Although cross subtype protection may be enhanced by bivalent or trivalent VHH, as was observed for RSV and Influenza, the most spectacular improvements were achieved with biparatopic VHH.

A poorly RSV B1 neutralizing VHH (RSV-E4) with high affinity for the RSV Long strain (subtype A) was combined with a VHH (RSV-D3) neutralizing the RSV Long strain (subtype A), but not the RSV B1 strain. The resulting biparatopic VHH construct had low nanomolar neutralizing potency against RSV B1 strain than the biparatopic construct, whereas the bivalent construct of the neutralizing VHH RSV-D3 had a 55-fold lower potency, indicating the recognition of two different epitopes may be an advantage for cross protection. The biparatopic construct consisting of RSV-D3 and RSV-E4 showed increased neutralization potency against the RSV Long strain as compared to the monovalent VHH. A shorter linker (9 or 15 residues) seemed to be preferred enforcing intramolecular binding to the D3 and E4 epitopes.

Compared to the monovalent VHHs, combining two neutralizers, Rab-E8 and Rab-H7 into biparatopic VHH increased the potency 1,572-fold against Rabies CVS-11, while the homo-bivalent constructs had around 20-fold increased potency, with IC_50_ similar to the mouse monoclonal antibody 8-2 [20]. The cross protection against Lyssavirus EBLV-1 was also considerably improved with this construct, being 147-fold better than the monovalent building blocks, again showing the power of targeting two different epitopes on a trimeric viral spike protein.

Influenza virus hemagglutinin is a trimeric spike protein used for attachment to the host cell receptor. The neutralizing VHH selected against Influenza H5N1 virus binds to the sialic acid binding site and thereby inhibits virus attachment to cells as observed both in the neutralization and Hemagglutination inhibition assay. Different constructs of IgG directed against trimeric spikes of Influenza and HIV have been compared, with IgG being more potent than its monovalent and bivalent derivates [26], [27]. From these studies it can be concluded that size, flexibility of the linkers and less occlusion effects are important factors to achieve potent multivalent molecules. VHHs are very small binding domains (15 kDa), which may allow penetration between the viral spikes, similar to what has been observed for enzymes with cleft recognition of the VHH [28]. Moreover, the small size of VHHs may reduce the risk of occlusions, which was observed for the larger antibody 4E10 constructs and thereby restricted access to the desired epitope by Klein et al [27].

For the flexibility, we used the Gly_4_/Ser linker between VHHs. This may allow binding and cross linking both intramolecularly to two of the three units of the viral spikes, intermolecularly to different spikes on a virus or by agglutination of the viruses. Furthermore, multimerization of VHHs may inhibit conformational changes of the viruses, thereby preventing fusion to host cell membranes. The intrinsic properties of the multimerized VHH were not investigated in this study, but previous reports on multimerized VHH show that the constructs are stable and stay intact in plasma at 37°C for a long time (44 hours) and the half life can be further improved by including an anti-albumin VHH in the multimeric construct [29], [7]. As reviewed by Saerens et al and Harmsen et al, the advantages of VHH over monoclonal antibodies are many such as the rapid tissue penetration, recognition of hidden antigenic sites, but the lack of effector functions and the decreased half-life is a disadvantage compared to monoclonal antibodies [10], [30]. Due the small size and high degree of human sequence homology of the frameworks of the VHH humanization processes to avoid immunogenicity seems straightforward and VHH with long serum half-life against RANKL has already successfully passed phase I clinical trial (Ablynx NV).

This is the first report of multivalent VHHs against trimeric spikes of virus causing infections in humans with greatly increased neutralization and cross protection potency.

## Materials and Methods

### Generation of viral specific Llama VHH

All animal experiments were conducted with the approval of the Ethical committee of the Faculty of Veterinary Medicine (University of Ghent, Belgium, EC number is 2006/076) and Ethical Committee of the IPH and the Veterinary and Agrochemical Research Centre (VAR, Brussels, Belgium, IPH authorization nr. LA1230177, advice nr. 070515-04). The animal immunization protocol is based upon on the guidelines available for Guanaco and Vicuña (llama species) as described in the Ministerial Decree of 05.03.1999 (for zoo animals) and the guidelines for farm animals used as laboratory animals described in Appendix A of the European convention for the protection of vertebrate animals used for experimental and other scientific purposes, from the European Treaty Series (ETS) 123.

Immunizations of llamas and library constructions were performed as described previously [3], [8] with six weekly injections for RSV and H5N1 and collection of 150 ml blood for isolation of RNA from the peripheral blood lymphocytes seven weeks after the initial immunization, resulting in two libraries for each viral target. The rabies immunizations were performed five times distributed over 57 days. The antigens used for immunizations were: trimeric RSV F_TM-_ protein (membrane anchor less form of the fusion protein, 70 kDa as monomeric protein [31]), H5N1 Hemagglutinin (H5, A/Vietnam/1203/2004, Protein Sciences Corporation) or Inactivated Rabies Vaccine Mérieux HDCV (genotype 1, Wistar Pitman Moore strain, Sanofi Pasteur MSD). Of the RSV F_TM-_ protein and of the H5 hemagglutinin, 40 µg were used for the first two doses and 20 µg for the following four doses.

RNA isolation and library constructions were performed as previously described amplifying the VHH genes and ligate them into a phagemid vector for display on phage resulting in libraries of size around 10^8^ [8].

Selections were performed on immobilized recombinant trimeric proteins and unspecific and competitive elutions using monoclonal antibodies were performed as previously described [3]. Synagis® (PalivizuMab, MedImmune Inc., humanized monoclonal IgG_1_κ antibody [16]) was used for competitive elution of RSV F_TM-_ protein specific VHH. Selections for Rabies glycoprotein (G protein) specific VHH were performed on 8 wells strips pre-coated with G protein from genotype 1, PV (Pasteur) strain (Platelia II Rabies plates, Bio-Rad Laboratories). Specific elutions were performed using a Rabies specific mouse monoclonal IgG_2a_ 8-2 (EBL-1, genotype 5 specific) [20]. For all three viruses, unspecific elutions of bound phage using trypsin were performed.

Eluted phage were used to infect exponentially growing *E. coli* TG1 that were plated on Luria broth (LB) agar plates containing 2% (w/v) glucose and 100 µg/ml ampicillin. Periplasmic extracts containing VHH were prepared according to standard protocols and selected VHH were sent for sequencing.

### Binding ELISA

To determine binding specificity to the viral coat proteins, periplasmic extracts and purified VHH were tested in ELISA binding assays. One hundred ng/ml RSV F_TM-_ protein, 0.5 µg/well RSV (Hytest, Turku, Finland) or 200 ng/ml Hemagglutinin H5 were immobilized on Nunc Maxisorp microtiter plates in PBS over night at 4°C. Rabies G protein pre-coated 8-well strips from BioRad were used for binding studies of Rabies specific VHH. Free binding sites were blocked using 4% skimmed milk or 1% casein in PBS (w/v) for 1–2 h at RT. Ten µl of VHH containing periplasmic fractions or dilutions of purified VHH in 100 µl were added to the blocked wells and allowed to bind for 1 h at RT. After incubation and washing steps, 1/2,000 rabbit anti-llama antibody (a kind gift from Unilever Research) and then 1/10,000 HRP-conjugated goat anti-rabbit antibody (DAKO) or 1/2,000 mouse anti-*c-myc* antibody and then 1/10,000 HRP-conjugated donkey anti-mouse antibody were added to detect binding of the VHH containing periplasmic fractions or purified VHH. Both antibodies were incubated for 1 h at RT. Peroxidase activity was developed with *o*-phenylenediamine (OPD) or TMB in the presence of H_2_O_2_ and the reactions were stopped with 1M H_2_SO_4_. Absorptions at 490 nm (OPD) or 450 nm (TMB) were measured using a microtiter plate reader. Competition was determined based on lower optical density (OD) values compared to controls having received no VHH or irrelevant VHH. The assays were repeated at least three times, but with some variation in secondary antibodies and read outs, confirming the binding pattern of the VHH.

### ELISA based competition assays

Competition assays were used for further characterization of the different anti-viral VHH.

For RSV specific VHH, RSV F_TM-_ (200 ng/well) were immobilized on Nunc Maxisorp microtiter plates in PBS over night at 4°C. Free binding sites were blocked using 4% skimmed milk or 2% bovine serum albumin in PBS (w/v). Ten or 20 µl VHH containing periplasmic fractions or dilutions of purified VHH were added together with 0.67 nM Synagis Mab, 3 nM Synagis Fab (HA-tagged, a kind gift from Ablynx NV) or 3 nM 101F Fab (HA-tagged, a kind gift from Ablynx NV). After incubation and a wash step, antibody binding was revealed using HRP-conjugated goat antibody specific for human Fc for Synagis Mab, a mouse antibody specific for HA-tag (Zymed laboratories) in combination with a HRP-conjugated rabbit antibody specific for mouse antibodies (Dako BV) for detection of Synagis Fab and 101F Fab.

For Influenza H5 specific VHH, fetuin from fetal calf serum (10 µg/well, Sigma-Aldrich) was immobilized on Nunc Maxisorp microtiter plates in PBS over night at 4°C. 100 ng/ml biotinylated H5 A/Vietnam/1194/04 (5 molar excess of sulfo-NHS-biotin incubated with the Influenza H5 for 30 min at RT before dialysis against PBS) was added to the coated wells and incubated with 10 or 20 µl of VHH containing periplasmic fractions or dilutions of purified VHH. After incubation for 1 h and washing steps, 1/5,000 diluted HRP-conjugated streptavidin was added for detection of biotinylated H5 and incubated for 1 h at RT.

For Rabies competition assays, Rabies G protein pre-coated 8-well strips from BioRad were used. Free binding sites were blocked using 4% skimmed milk or 2% bovine serum albumin in PBS (w/v). Next, 4 nM mouse IgG_2a_ monoclonal 8-2 was added to the coated wells and incubated with 10 or 20 µl of VHH containing periplasmic fractions or dilutions of purified VHH. Control periplasmic fractions or VHH selected against other viral coat proteins were included. After incubation and a wash step, antibody binding was revealed using a HRP conjugated donkey antibody specific for mouse antibodies for detection of mouse monoclonal IgG_2a_ 8-2.

For all viral competition assays, peroxidase activity was developed with *o*-phenylenediamine (OPD) or TMB in the presence of H_2_O_2_ and the reactions were stopped with 1M H_2_SO_4_. Absorptions at 490 nm (OPD) or 450 nm (TMB) were measured using a microtiter plate reader. Competition was determined based on lower optical density (OD) values compared to controls having received no VHH or irrelevant VHH.

### Reactivity of VHH against RSV escape mutants

The isolation and characterization of RSV escape mutants has been described [15]. Hep-2 cells were infected with the different viruses at m.o.i of 0.1 pfu/cell, harvested 72 h later, when cytopathic effect was maximal, and extracts were made as described [32]. Dilutions of the extracts were tested by ELISA with a polyclonal serum against F [32] to normalize the amount of F protein in each extract. Then, an equal amount of F protein from the different mutants was tested for reactivity with non-saturating amounts of VHHs, 0.2 µg/ml in ELISA. Absorbance results were normalized for reactivity on the reference virus strain (Long wild type) strain as well as on the control VHH RSV-C7.

### Surface Plasmon resonance for affinity measurements

For affinity measurements of VHH against Influenza H5 and epitope mapping, 2,000 Resonance units (RU) of Influenza H5 was coupled on a Sensorchip CM5 in 10 mM sodium acetate pH 5.5 and immobilized by aminecoupling (aminecoupling kit, BIAcore) and run in a BIAcore 3000. Dilutions of the VHH were added at concentrations 12.5–250 nM and run over a reference flow channel with no H5 and run in parallell over the H5 coupled flow channel at a flow rate of 30 µl/min. One mM NaOH was used for regeneration of the chip.

Affinity measurements of VHH against RSV F_TM-_ were performed in a BIAcore T1000. Around 400 RU RSV F_TM-_ were coupled in 10 mM sodium acetate buffer pH 4.5 and immobilized by aminecoupling. Dilutions of the VHH were added at concentrations 1–243 nM for the VHH and 0.5–500 nM for the Synagis Mab and Fab and run over a reference flow channel with no RSV F_TM-_ and run in parallell over the RSV F_TM-_ coupled flow channel at a flow rate of 45 µl/min. Onehundred mM HCl was used for regeneration of the chip.

Evaluation of association rate constants (*k_on_*), dissociation rate constants (*k_off_*) and equilibrium dissociation constants (*K_D_* = *k_on_*/*k_off_*) was performed by fitting a 1∶1 interaction model (Langmuir binding model), removing the background from the reference flow channel by Biacore T100 software v1.1 for RSV evaluations and Biacore 3000 Software v4.1 for the Influenza H5 evaluations.

### Re-cloning and purification of monovalent and multimeric VHH

The encoding sequences for selected VHH were re-cloned in expression vectors containing C-terminal *c-myc* and His_6_ tags, pAX051 or pAX100. Generation of multimeric constructs were performed as previously described [8]. Briefly, bivalent or biparatopic constructs connected by Gly_4_/Ser linkers of different lengths and composition were generated by separate PCR reactions (one for the N-terminal and one for the C-terminal VHH subunit and for trivalent constructs, one extra PCR reaction for the middle VHH subunit) using different sets of primers encompassing parts of the linker and restriction sites *Sfi*I in the N-terminal, *Bst*EII in the C-terminal and *Bsp*EI, for re-cloning into the expression vectors. The Gly/Ser linkers were ranging from 9 (G_4_SG_3_S) to 35 (G_4_S)_7_ residues. The biparatopic constructs (fusion of two different VHH) were done in both directions.

After induction of a logarithmic phase culture with 1 mM isopropyl-β-D-thiogalactopyranoside (IPTG) for expression of His_6_-tagged proteins, VHH were purified from the periplasmic fraction by Immobilized affinity chromatography (IMAC) using Talon metalaffinity resin (BD Biosciences) and washed and eluted according to the manufacturer's protocol. The eluted VHH were extensively dialyzed against PBS and stored at −20°C in small aliquots.

To check the size and purity of the purified VHH fragments, 0.5–1 µg was run on 15% SDS-PAGE under reducing conditions and stained with Coomassie Brilliant Blue (CBB).

### RSV micro neutralization assay

The potency of purified VHH in neutralization of different type A and B RSV strains was tested by the *in vitro* micro neutralization assay. Viral stocks of RSV Long strain (originally isolated in Baltimore, MD, 1956) and RSV B1 (ATCC VR-1580) were prepared in HEp-2 cells and subsequently titrated to determine the optimal infectious dose (1–3 pfu/cell) for use in the micro neutralization assay. HEp-2 cells were seeded at a density of 1.5×10^4^ cells/well into 96-well plates in DMEM medium containing 10% fetal calf serum (FCS) supplemented with penicillin and streptomycin (100 U/ml and 100 µg/ml, respectively) and incubated for 24 h at 37°C in a 5% CO_2_ atmosphere. The RSV strains were pre-incubated with serial dilutions of purified VHH in a total volume of 50 µl for 30 min at 37°C. The medium of the HEp-2 cells was replaced with the premix to allow infection for 2 h, after which 0.1 ml of assay medium was added. Cells were incubated for additional 72 h at 37°C in a 5% CO_2_ atmosphere, after which cells were fixed with 80% cold acetone (Sigma-Aldrich) in PBS (100 µl/well) for 20 min at 4°C and left to dry completely. The presence of the F-protein on the cell surface was detected in an ELISA type assay. Fixed HEp-2 cells were blocked with 2% BSA solution in PBS for 1 h at RT, incubated with for 1 h with polyclonal rabbit serum specific for F-protein or monoclonal antibody Synagis (2 µg/ml). HRP conjugated goat antibody specific for rabbit or HRP conjugated goat antibody specific for Human IgG (Fcγ fragment, Jackson ImmunoResearch) were used for detection, after which the ELISA was developed according to standard procedures. RSV-D3, Synagis Mab, Synagis Fab and irrelevant VHH were included as controls. The percentage competition was calculated based on controls receiving irrelevant VHH (0%) and no virus (100%) using GraphPad Prism and non-linear regression curve fit. All experiments were repeated two times.

### MLV(H5) pseudotype neutralization assay

The neutralizing capacity of VHH against Influenza H5 was evaluated in the MLV(H5) Pseudotyped neutralization assay described by Temperton et al, 2007 [16]. The pseudotyped virus as used in this study were expressing the Influenza hemagglutinin H5 of A/Vietnam/1194/04 (Clade 1), A/Vietnam/1203/04 (Clade 1, a kind gift from Barbara Capecchi, Novartis Vaccines, Siena), A/turkey/Turkey/1/05 (Clade 2.2, a kind gift from Yipu Lin, MRC National Institute for Medical research, Mill Hill, UK), A/Anhui/1/05 (Clade 2.3.4, a kind gift from Barbara Capecchi), A/Bar-headed goose/Qinghai/1A/05 (Clade 2.2), A/Whooping swan/Mongolia/244/05 (Clade 2.2, a kind gift from Barbara Capecchi) and A/chicken/Korea/ES/03 (Clade 2.5, a kind gift from Chung Kang, Korea CDC).

Briefly, purified VHH were twofold serially diluted in culture medium, and mixed with MLV (H5) virions (10,000 RLU for Luciferase) at a 1∶1 v/v ratio. After incubation at 37°C for 1 h, 1×10^4^ 293T cells were added to each well of a 96-well flat-bottomed plate. Relative light units (RLU) for Luc were evaluated 48 h later by luminometry using the Promega Bright-Glo system (Promega) according to the manufacturer's instructions. The neutralizing VHH titers were determined as the concentration resulting in a 50% reduction of infection (as measured by marker gene transfer) compared with a pseudotype virus only control. For Luc, titers <100 were designated negative.

All samples were run in duplicates and the geometric mean was calculated. A standard serum (sheep H5 reference sera from NIBSC) was always included.

### Influenza microneutralization assay

The assay was based on the protocol recommended by WHO (2002) and adapted as follows: Two-fold serial dilutions of VHHs (initial concentration 5 µM) were mixed 1∶1 with 50 µl of NIBRG-14 (H5N1), PR8 (H1N1) or X47 (H3N2) virus and incubated at 37°C for 2 h after which the mixture was transferred to MDCK cell monolayers and incubated for 18 to 22 h at 37°C in virus growth medium with trypsin. The amount of virus used in the assays was 100 tissue culture infectious doses as determined by the same NP (nucleoprotein)-ELISA used to determine microneutralization efficacy. Anti-NP antibody was obtained through the NIH Biodefense and Emerging Infections Research Resources Repository, NIAID, NIH: Antisera Panel to Isolated Antigens of Influenza Virus, NR-10208. After fixation of the cells with ethanol∶acetone (1∶1) for 20 min at −20°C, influenza virus growth was detected by an antibody specific for NP. The fixed plates were washed with PBS containing 0.05% Tween 20. A goat antibody specific for NP was diluted 3,000-fold in PBS containing 1% bovine serum albumin and 0.1% Tween 20 and incubated for 1 h. Horseradish peroxidase-labelled goat antibody was diluted 4,000-fold and incubated for 1 h at room temperature. One hundred microliters of freshly prepared HRP substrate was added to each well, and the plates were incubated at room temperature for approximately 5 min. The reaction was stopped with an equal volume of 1 N sulfuric acid. The absorbance was measured at 450 nm and 595 nm with an iMark microplate reader (Bio-rad).

### Hemagglutination Inhibition assay

The assay was based on the protocol from WHO (http://www.who.int/vaccine_research/diseases/influenza/WHO_manual_on_animal-diagnosis_and_surveillance_2002_5.pdf). After determining the optimal HA units for the Influenza H5N1, NIBRG-14 carrying the A/Vietnam/1194/2004, two-fold dilutions of purified VHH (initial concentration 5 µM) were added to 4 HA units of NIBRG-14 and 1% chicken red blood cells (RBC) in PBS (total volume of 25 µl) in 96-well V-shaped microtiter plates. The minimal inhibitory concentration of hemagglutination for the different VHH constructs compared to control VHH was determined.

### Rabies virus neutralization assays

Two assays were used to examine virus neutralization, depending on whether the virus was propagated in cell culture or in the brain of mice upon intracerebral inoculation. Cell culture-grown viruses were examined in the Rapid Fluorescent Focus Inhibition Test (RFFIT) and brain-grown viruses in an alternative infection assay with neuroblastoma N2a cells.

Neutralizing potency against 4 cell culture-grown viruses was examined by RFFIT. CVS-11 (Challenge Virus Standard-11, The American Type Culture Collection (ATCC) reference VR-959) is a virulent genotype 1 strain, which is used as the reference laboratory strain in the RFFIT. ERA (Evelyn-Rotnycki-Abelseth) is an attenuated genotype 1 virus which is used as an oral vaccine for immunization of wildlife (ATCC VR322). CB-1 (Chien Beersel) is a virulent genotype 1 isolate from the brain of a rabid dog from Morocco [33]. The EBLV-1 (European Bat Lyssavirus-1) strain 8919FRA is a virulent genotype 5 strain, which was isolated from an *Eptesicus serotinus* bat in France [34] and kindly provided by Dr. L. Dacheux from the Pasteur Institute of Paris. The viral stocks were grown in Baby Hamster Kidney-21 cells (BHK-21) cells, except for CB-1, which was grown in neuroblastoma N2a cells. The lysates of infected cell cultures were centrifuged at 20,000*g* for 20 min at 4°C and supernatants were stored at −80°C.

The RFFIT was performed according to the Manual of Diagnostic Tests and Vaccines for Terrestrial Animals (Office International des Epizooties, 2008). Briefly, susceptible cells (BHK-21 for CVS-11, ERA, EBLV-1 and neuroblastoma N2a cells for CB-1) were infected with a standard amount of virus after pre-incubation with VHH. The virus neutralization was quantified by counting the reduction in nucleocapsid-positive cells upon staining with fluorescein isothiocyanate (FITC)-coupled specific rabies monoclonal antibodies (Fujirebio Diagnostics, Inc.). In each test, one OIE canine reference serum, containing 0.50 IU/ml, and two WHO human reference sera, containing respectively 0.50 IU/ml and 6.00 IU/ml, were included as controls of the assays. (International Units (IU)/ml in reference to “The Second International standard for Anti-Rabies Immunoglobulin” purchased from the United Kingdom National Institute for Biological Standards and Control.) All experiments were performed in triplicates and the mean was calculated. Neutralizing potency of the VHH was expressed as mean nM IC_50_ (VHH concentration needed to neutralize 50% of 10^3^ TCID_50_ of CVS-11 on BHK cells). According to the WHO convention, a serum titer of 0.50 IU/ml is protective *in vivo*. Neutralizing potency of the other strains was defined in Equivalent Units (EU)/ml, which correspond closely to IU/ml. Mab 8-2 is a mouse monoclonal IgG2_a_ raised against the rabies glycoprotein G [20].

Seven brain-grown genotype 1 viruses were tested in a neuroblastoma N2a infection assay [35]. All viruses were provided by Dr. L. Dacheux from the Pasteur Institute of Paris. Six viruses were wild isolates, among which an isolate from a dog from Cambodia (9912CBG, accession nr. EU086169/EU086132), a fox from France (9147FRA, accession nr. EU293115), a raccoon dog from Poland (9722POL), a human patient from Thailand (8740THA), a dog from the Ivory Coast (07059IC, accession nr. EU853615/FJ545659) and a dog from Niger (9009NIG, accession nr. EU853646). One virus was the laboratory strain CVS IP13. Briefly, suspensions of infected brain tissues (0.1% w/v) were prepared in cell culture medium at 4°C. Ten-fold dilutions of the infected brain suspensions were pre-incubated with about 0.2 µM of VHH for 90 min at 37°C and 5% CO_2_. Then, susceptible neuroblastoma N2a cells were added to the mix. Two days later, the number of nucleocapsid-positive cells was counted upon staining with FITC-coupled specific rabies monoclonal antibodies (Fujirebio Diagnostics, Inc.). Neutralization was defined as a minimum 100-fold reduction of virus infectivity in the brain after preincubation with antibody (Mab 8-2) or VHH compared to a control VHH (NR4 against RSV).
